# Crystal structure, Hirshfeld surface analysis and inter­action energy and DFT studies of 5,5-diphenyl-1,3-bis­(prop-2-yn-1-yl)imidazolidine-2,4-dione

**DOI:** 10.1107/S2056989019007801

**Published:** 2019-06-04

**Authors:** Ismail Ghandour, Abdelouahed Bouayad, Tuncer Hökelek, Amal Haoudi, Frédéric Capet, Catherine Renard, Youssef Kandri Rodi

**Affiliations:** a Laboratoire de Chimie de la Matière Condensée, Université Sidi Mohamed Ben Abdallah, Faculté des Sciences et Techniques, Route d’Immouzzer, BP 2202, Fez, Morocco; b Department of Physics, Hacettepe University, 06800 Beytepe, Ankara, Turkey; c Laboratoire de Chimie Organique Appliquée, Université Sidi Mohamed Ben Abdallah, Faculté des Sciences et Techniques, Route d’Immouzzer, BP 2202, Fez, Morocco; d Unité de Catalyse et de Chimie du Solide (UCCS), UMR 8181, Ecole Nationale Supérieure de Chimie de Lille, Université Lille 1, 59650 Villeneuve, d’Ascq, Cedex, France

**Keywords:** crystal structure, imidazolidine, oxazole, π-stacking, Hirshfeld surface

## Abstract

The title compound, C_21_H_16_N_2_O_2_, consists of an imidazolidine unit linked to two phenyl rings and two prop-2-yn-1-yl moieties. The imidazolidine ring is oriented at dihedral angles of 79.10 (5) and 82.61 (5)° with respect to the phenyl rings, while the dihedral angle between the two phenyl rings is 62.06 (5)°. In the crystal, inter­molecular C—H_Prop_⋯O_Imdzln_ (Prop = prop-2-yn-1-yl and Imdzln = imidazolidine) hydrogen bonds link the mol­ecules into infinite chains along the *b*-axis direction. Two weak C—H_Phen_⋯π inter­actions are also observed. The Hirshfeld surface analysis of the crystal structure indicates that the most important contributions for the crystal packing are from H⋯H (43.3%), H⋯C/C⋯H (37.8%) and H⋯O/O⋯H (18.0%) inter­actions. Hydrogen bonding and van der Waals inter­actions are the dominant inter­actions in the crystal packing. Computational chemistry indicates that the C—H_Prop_⋯O_Imdzln_ hydrogen-bond energy in the crystal is −40.7 kJ mol^−1^. Density functional theory (DFT) optimized structures at the B3LYP/6–311G(d,p) level are compared with the experimentally determined mol­ecular structure in the solid state. The HOMO–LUMO behaviour was elucidated to determine the energy gap.

## Abstract 2

The title mol­ecule consists of an imidazolidine unit linked to two phenyl rings and two prop-2-yn-1-yl moieties. The imidazolidine ring is oriented at dihedral angles of 79.10 (5) and 82.61 (5)° with respect to the phenyl rings, while the dihedral angle between the two phenyl rings is 62.06 (5)°. In the crystal, C—H_Prop_⋯O_Imdzln_ (Prop = prop-2-yn-1-yl and Imdzln = imidazolidine) hydrogen bonds link the mol­ecules into infinite chains along the *b-*axis direction. Two weak C—H_Phen_⋯π inter­actions are also observed.

## Chemical context   

Pyrazolo­nes are an important class of heterocyclic compounds that occur in many drugs and their derivatives have long been of inter­est to medicinal chemists for their wide range of biological activities (Pawar & Patil, 1994 ▸), including anti­bacterial, anti­diabetic, immunosuppressive agents, and substances displaying hypoglycemic, anti­viral and anti­neoplastic actions (Pathak & Bahel, 1980 ▸; Naik & Malik, 2010 ▸; Srivalli *et al.*, 2011 ▸). Their pharmaceutical applications include use as a non-steroidal anti-inflammatory agent in the treatment of arthritis and other musculoskeletal and joint disorders (Amir & Kumar, 2005 ▸), and as analgesic, anti­pyretic (Badawey & El-Ashmawey, 1998 ▸) and hypoglycemic agents (Das *et al.*, 2008 ▸). They also have fungicidal (Singh & Singh, 1991 ▸) and anti­microbial properties (Sahu *et al.*, 2007 ▸), and some have been tested as potential cardiovascular drugs (Higashi *et al.*, 2006 ▸). In the past few years, research has been focused on existing mol­ecules and their modifications in order to reduce side effects and to explore other pharmacological and biological activity (Sahu *et al.*, 2007 ▸; Naik & Malik, 2010 ▸; Srivalli *et al.*, 2011 ▸). As a continuation of our research on the development of new N-substituted pyrazolone derivatives and the evaluation of their potential pharmacological activities, we report herein the synthesis, the mol­ecular and crystal structures, the Hirshfeld surface analysis and inter­molecular inter­action energies and density functional theory (DFT) computational calculation of the title compound, (I).
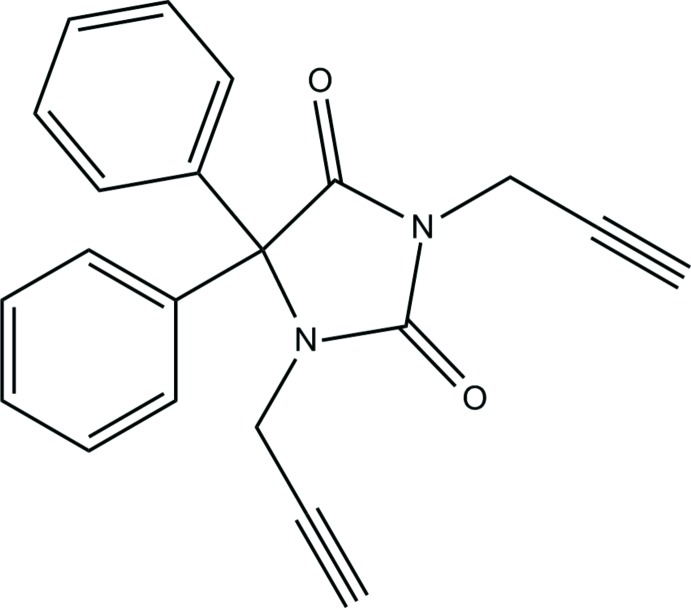


## Structural commentary   

**Figure 1:**
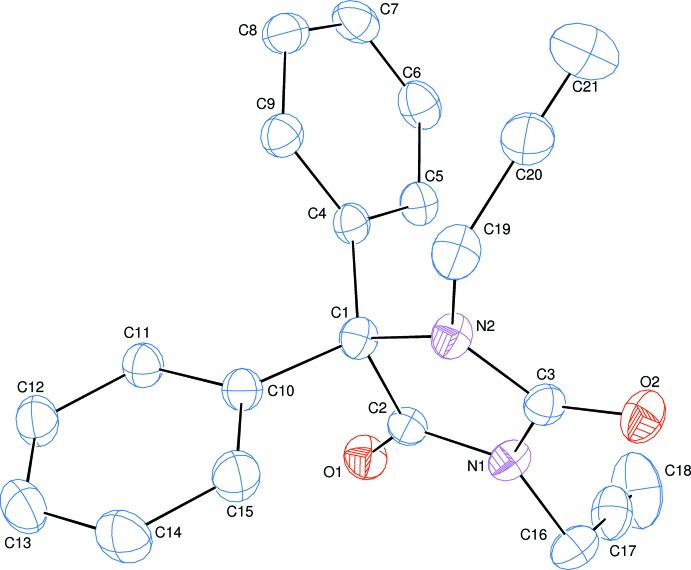
The mol­ecular structure of the title compound with the atom-numbering scheme. Displacement ellipsoids are drawn at the 50% probability level.

The title mol­ecule consists of an imidazolidine unit linked to two phenyl rings and two prop-2-yn-1-yl moieties (Fig. 1 ▸). The planar five-membered imidazolidine ring, *A* (N1/N2/C1–C3), is oriented at dihedral angles of 79.10 (5) and 82.61 (5)°, respectively, to phenyl rings *B* (C4–C9) and *C* (C10–C15), while the dihedral angle between the two phenyl rings is 62.06 (5)°. Atoms O1, O2, C16 and C19 are at distances of 0.0271 (12), −0.1040 (12), 0.1657 (19) and −0.0336 (19) Å from the mean plane of the imidazolidine ring, *A*. The orientation of the prop-2-yn-1-yl moieties with respect to the imidazolidine unit can be described by the C3—N1—C16—C17 and C3—N2—C19—C20 torsion angles of −115.3 (2) and 76.6 (2)°, respectively.

## Supra­molecular features   

**Figure 2:**
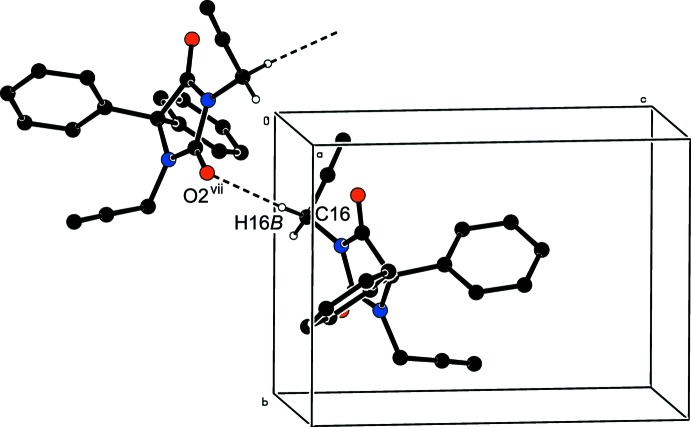
A partial packing diagram viewed down the *a*-axis direction. C—H_Prop_⋯N_Imdzln_ (Prop = prop-2-yn-1-yl and Imdzln = imidazolidine) hydrogen bonds (Table 1 ▸) are shown as dashed lines. Symmetry code: (vii) 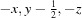.

In the crystal, C—H_Prop_⋯O_Imdzln_ (Prop = prop-2-yn-1-yl and Imdzln = imidazolidine) hydrogen bonds (Table 1 ▸ and Fig. 2 ▸) link the mol­ecules into infinite chains along the *b*-axis direction. Two weak C—H_Phen_⋯π inter­actions (Table 1 ▸) may also contribute to the stabilization of the crystal structure.

## Hirshfeld surface analysis   

**Figure 3:**
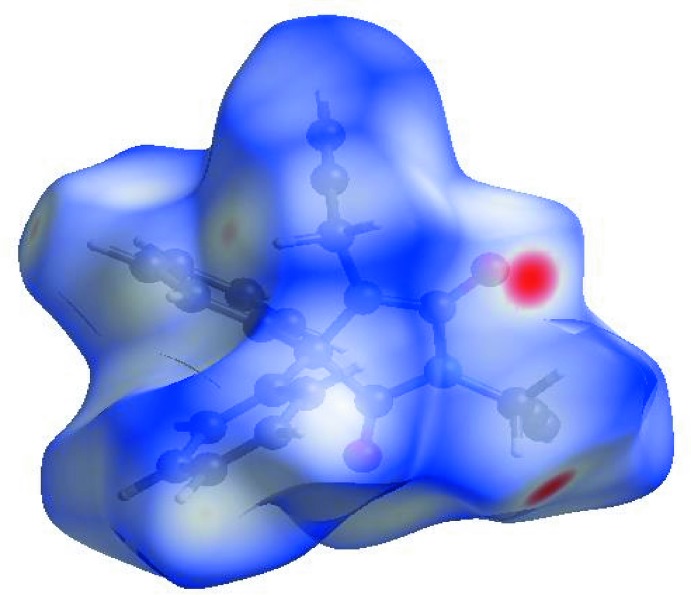
View of the three-dimensional Hirshfeld surface of the title compound plotted over *d*_norm_ in the range −0.2703 to 1.2169 a.u.

**Figure 4:**
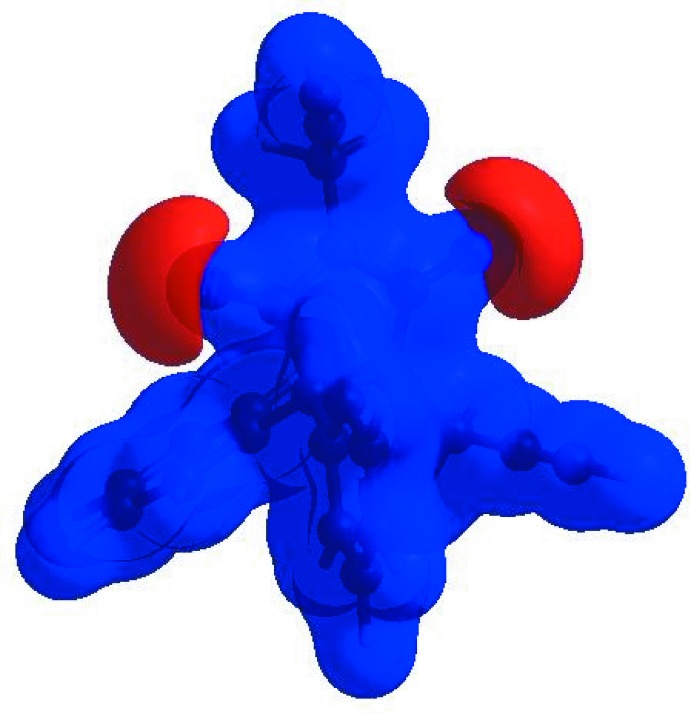
View of the three-dimensional Hirshfeld surface of the title compound plotted over electrostatic potential energy in the range −0.0500 to 0.0500 a.u. using the STO-3 G basis set at the Hartree–Fock level of theory. Hydrogen-bond donors and acceptors are shown as blue and red regions around the atoms corresponding to positive and negative potentials, respectively.

**Figure 5:**
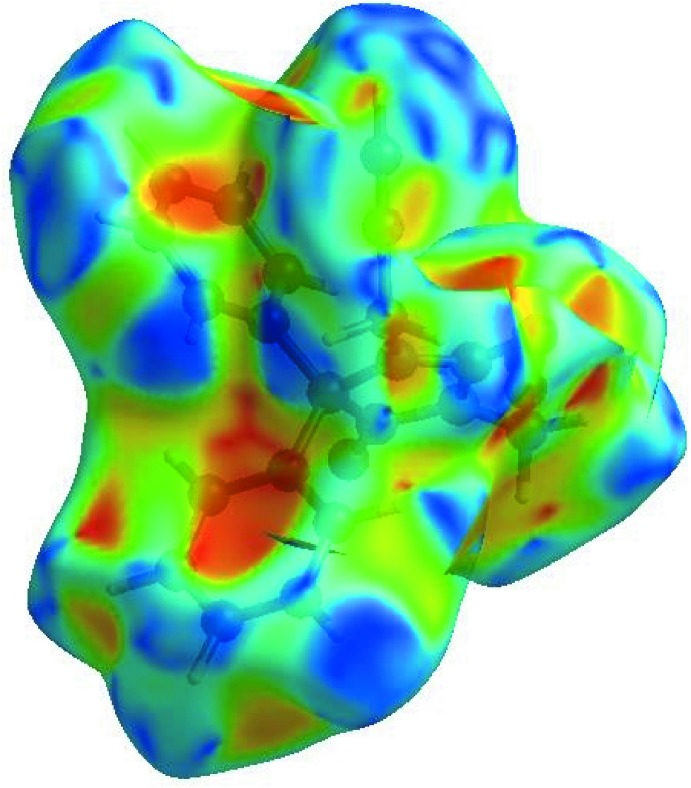
Hirshfeld surface of the title compound plotted over shape-index.

In order to visualize the inter­molecular inter­actions in the crystal of the title compound, a Hirshfeld surface (HS) analysis (Hirshfeld, 1977 ▸; Spackman & Jayatilaka, 2009 ▸) was carried out by using *CrystalExplorer17.5* (Turner *et al.*, 2017 ▸). In the HS plotted over *d*_norm_ (Fig. 3 ▸), the white surface indicates contacts with distances equal to the sum of van der Waals radii, and the red and blue colours indicate distances shorter (in close contact) or longer (distinct contact) than the van der Waals radii, respectively (Venkatesan *et al.*, 2016 ▸). The bright-red spots appearing near O2 and hydrogen atom H16*B* indicate their roles as the respective donors and/or acceptors; they also appear as blue and red regions corresponding to positive and negative potentials on the HS mapped over electrostatic potential (Spackman *et al.*, 2008 ▸; Jayatilaka *et al.*, 2005 ▸) as shown in Fig. 4 ▸. The blue regions indicate the positive electrostatic potential (hydrogen-bond donors), while the red regions indicate the negative electrostatic potential (hydrogen-bond acceptors). The shape-index of the HS is a tool to visualize the π–π stacking by the presence of adjacent red and blue triangles; if there are no adjacent red and/or blue triangles, then there are no π–·π inter­actions. Fig. 5 ▸ clearly suggest that there are no π–π inter­actions in (I).

**Figure 6:**
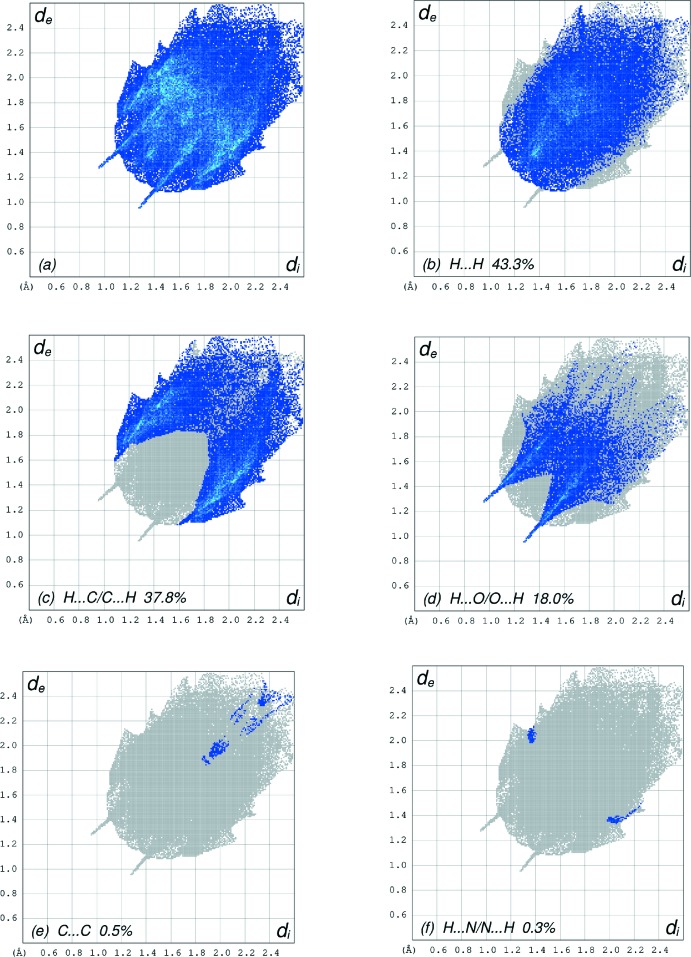
The full two-dimensional fingerprint plots for the title compound, showing (*a*) all inter­actions, and delineated into (*b*) H⋯H, (*c*) H⋯C/C⋯H, (*d*) H⋯O/O⋯H, (*e*) C⋯C and (*f*) H⋯N/N⋯H inter­actions. The *d*_i_ and *d*_e_ values are the closest inter­nal and external distances (in Å) from given points on the Hirshfeld surface contacts.

The overall two-dimensional fingerprint plot, Fig. 6 ▸ *a*, and those delineated into H⋯H, H⋯C/C⋯H, H⋯O/O ⋯ H, C⋯C and H⋯N/N⋯H contacts (McKinnon *et al.*, 2007 ▸) are illustrated in Fig. 6 ▸ *b*–*f*, together with their relative contributions to the Hirshfeld surface while details of the various contacts are given in Table 2 ▸. The most important inter­action is H⋯H contributing 43.3% to the overall crystal packing, which is reflected in Fig. 6 ▸ *b* as widely scattered points of high density due to the large hydrogen content of the mol­ecule with the tip at *d*_e_ + *d*_i_ ∼2.44 Å. In the presence of two weak C—H⋯π inter­actions, the pair of the scattered points of wings resulting from H⋯C/C⋯H contacts, with a 37.8% contribution to the HS, have a symmetrical distribution of points, Fig. 6 ▸ *c*, with the thin edges at *d*_e_ + *d*_i_ = 2.67 Å. The fingerprint plot for H⋯O/O⋯H contacts (18.0% contribution), Fig. 6 ▸ *d*, has a pair of spikes with the tips at *d*_e_ + *d*_i_ = 2.24 Å.

**Figure 7:**
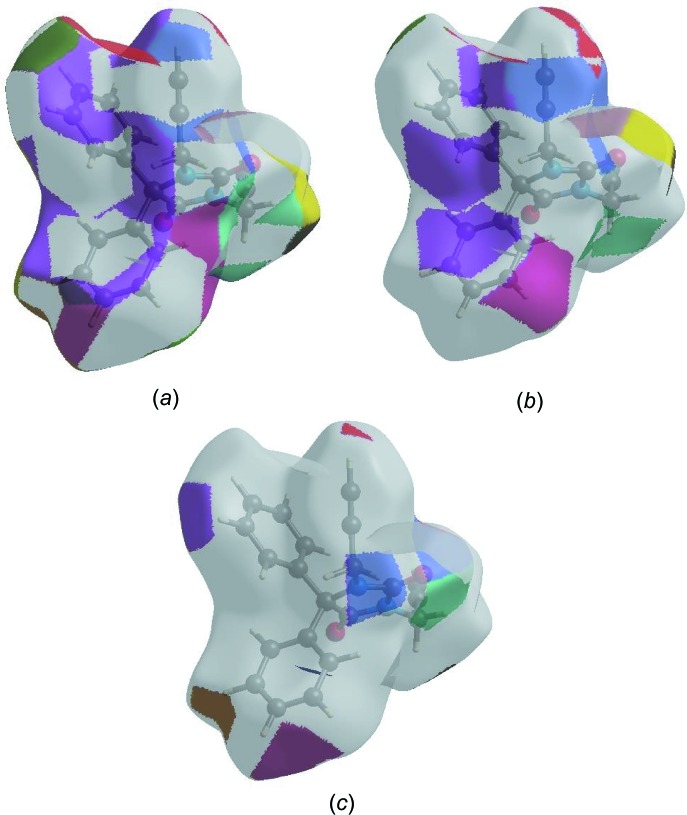
The Hirshfeld surface representations with the function *d*_norm_ plotted onto the surface for (*a*) H⋯H, (*b*) H⋯C/C⋯H and H⋯O/O ⋯ H inter­actions.

The Hirshfeld surface representations with the function *d*_norm_ plotted onto the surface are shown for the H⋯H, H⋯C/C⋯H and H⋯O/O⋯H inter­actions in Fig. 7 ▸ *a*–*c*.

The Hirshfeld surface analysis confirms the importance of H-atom contacts in establishing the packing. The large number of H⋯H, H⋯C/C⋯H and H ⋯ O/O⋯H inter­actions suggest that van der Waals inter­actions and hydrogen bonding play the major roles in the crystal packing (Hathwar *et al.*, 2015 ▸).

## Inter­action energy calculations   

The inter­molecular inter­action energies (Table 3 ▸) were calculated using the CE–B3LYP/6–311G(d,p) energy model available in *CrystalExplorer17.5* (Turner *et al.*, 2017 ▸), where a cluster of mol­ecules is generated by applying crystallographic symmetry operations with respect to a selected central mol­ecule within a default radius of 3.8 Å (Turner *et al.*, 2014 ▸). The total inter­molecular energy (*E*_tot_) is the sum of electrostatic (*E*_ele_), polarization (*E*_pol_), dispersion (*E*_dis_) and exchange-repulsion (*E*_rep_) energies (Turner *et al.*, 2015 ▸) with scale factors of 1.057, 0.740, 0.871 and 0.618, respectively (Mackenzie *et al.*, 2017 ▸). The hydrogen-bonding inter­action energy (in kJ mol^−1^) was calculated to be −15.3 (*E*_ele_), −3.2 (*E*_pol_), −52.2 (*E*_dis_), 37.6 (*E*_rep_) and −40.7 (*E*_tot_) for the C—H_Prop_⋯N_Imdzln_ inter­action.

## DFT calculations   

**Figure 8:**
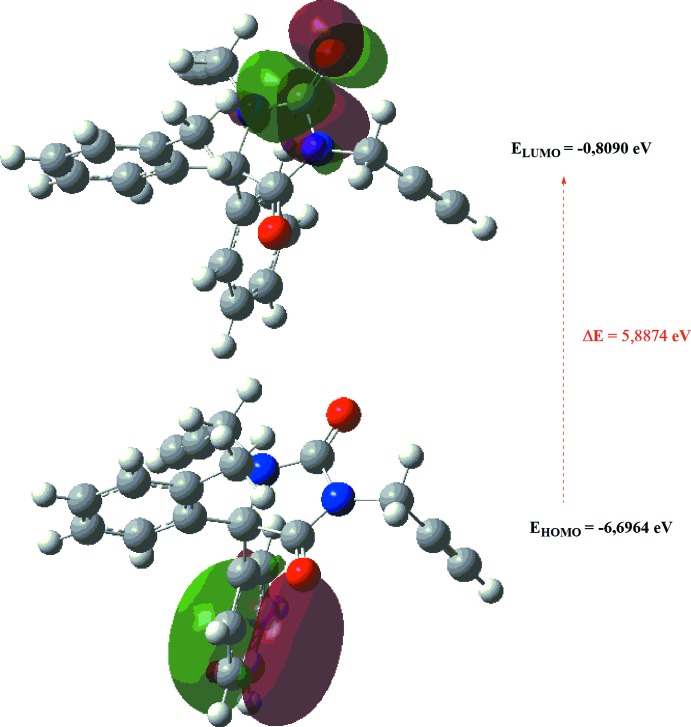
The energy band gap of the title compound.

The optimized structure of the title compound in the gas phase was generated theoretically *via* density functional theory (DFT) calculations using the standard B3LYP functional and 6–311G(d,p) basis set (Becke, 1993 ▸) as implemented in *GAUSSIAN 09* (Frisch *et al.*, 2009 ▸). The theoretical and experimental results are in good agreement (Table 4 ▸). The highest occupied mol­ecular orbital (HOMO), acting as an electron donor, and the lowest unoccupied mol­ecular orbital (LUMO), acting as an electron acceptor, are very important parameters for quantum chemistry. When the energy gap is small, the mol­ecule is highly polarizable and has high chemical reactivity. The DFT calculations provide some important information on the reactivity and site selectivity of the mol­ecular framework. *E*_HOMO_ and *E*_LUMO_ clarify the inevitable charge-exchange collaboration inside the studied material; the electronegativity (χ), hardness (η), potential (μ), electrophilicity (ω) and softness (*σ*) are recorded in Table 3 ▸. The significance of η and *σ* is to evaluate both the reactivity and stability of a compound. The electron transition from the HOMO to the LUMO energy level is shown in Fig. 8 ▸. The HOMO and LUMO are localized in the plane extending from the whole 5,5-diphenyl-1,3-di(prop-2-yn-1-yl)imidazolidine-2,4-dione ring. The energy band gap [Δ*E* = *E*_LUMO_ - *E*_HOMO_] of the mol­ecule is about 5.8874 eV, and the frontier mol­ecular orbital energies, *E*_HOMO_ and *E*_LUMO_ are −6.6964 and −0.8090 eV, respectively.

## Database survey   

A non-alkyl­ated analogue, namely 5,5-di­phenyl­imidazolidine-2,4-dione, has been reported (Camerman & Camerman, 1971 ▸), as well as three similar structures, 3-*n*-pentyl-5,5-di­phenyl­imidazolidine-2,4-dione (Guerrab *et al.*, 2017 ▸), 3-benzyl-5,5 di­phenyl­imidazolidine-2,4-dione (Guerrab *et al.*, 2018 ▸) and 3-[2-(4-fluoro­phen­yl)-2-oxoeth­yl]-5,5 di­phenyl­imidazolidine-2,4-dione (Mague *et al.*, 2014 ▸).

## Synthesis and crystallization   

The appropriate bromide propargil (2.4 ml, 20.0 mmol) was added to a solution of 5,5 di­phenyl­hydantoin (3.52 g, 10.0 mmol) in DMF (50 ml), potassium carbonate (2.76 g, 20.0 mmol) and tetra-*n*-butyl­ammonium bromide (0.32 g, 1.0 mmol) at room temperature. The reaction was monitored using TLC. After removal of the inorganic salt by filtration, the solution was evaporated under reduced pressure. The residue was separated by chromatography on a column of silica gel with ethyl acetate–hexane (*v*:*v* 3:7) as eluent. The isolated solid was crystallized from ethanol solution to afford colourless crystals (yield: 82%).

## Refinement   

Crystal data, data collection and structure refinement details are summarized in Table 5 ▸. Hydrogen atoms were located in a difference-Fourier map, and refined freely. The Flack absolute structure parameter (Parsons *et al.*, 2013 ▸) was refined; expected values are 0 for the correct and +1 for the inverted absolute structure. The refined value is −0.3 (4) (Sheldrick, 2015*b* ▸). Since the large e.s.d. means that the assignment is not unambiguous, the absolute structure was not determined reliably.

## Supplementary Material

Crystal structure: contains datablock(s) I, global. DOI: 10.1107/S2056989019007801/lh5908sup1.cif

Structure factors: contains datablock(s) I. DOI: 10.1107/S2056989019007801/lh5908Isup2.hkl

Supporting information file. DOI: 10.1107/S2056989019007801/lh5908Isup3.cdx

Supporting information file. DOI: 10.1107/S2056989019007801/lh5908Isup4.cml

CCDC reference: 1919743

Additional supporting information:  crystallographic information; 3D view; checkCIF report

## Figures and Tables

**Table 1:** Hydrogen-bond geometry (Å, °) *Cg*1 and *Cg*2 are the centroids of the C4–C9 and C10–C15 rings, respectively.

*D*—H⋯*A*	*D*—H	H⋯*A*	*D*⋯*A*	*D*—H⋯*A*
C16—H16*B*⋯O2^vii^	0.98 (3)	2.33 (3)	3.178 (3)	144 (2)
C9—H9⋯*Cg*1^vi^	0.93 (3)	2.93 (2)	3.778 (2)	152.7 (17)
C14—H14⋯*Cg*2^viii^	1.00 (3)	2.87 (2)	3.762 (3)	149.6 (17)

Symmetry codes: (vi) 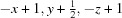; (vii) 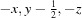; (viii) 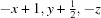.

**Table 2:** Selected interatomic distances (Å)

O1⋯H16*B*	2.84 (2)	C4⋯H11	2.679 (18)
O1⋯H13^i^	2.61 (2)	C6⋯H9^ii^	2.97 (2)
O1⋯H5	2.767 (18)	C6⋯H18^v^	2.90 (4)
O1⋯H8^ii^	2.62 (2)	C8⋯H11^vi^	2.94 (2)
O2⋯H18^iii^	2.68 (5)	C8⋯H19*A* ^ii^	2.83 (2)
O2⋯H19*B*	2.65 (2)	C9⋯H11	2.73 (2)
O2⋯H16*A*	2.50 (2)	C10⋯H9	2.75 (2)
O2⋯H16*B* ^iv^	2.33 (2)	C10⋯H19*A*	2.96 (2)
N2⋯H15	2.53 (2)	C11⋯H14^i^	2.94 (2)
C4⋯C20	3.372 (3)	C11⋯H9	2.79 (2)
C9⋯C19	3.378 (3)	C12⋯H14^i^	2.91 (2)
C9⋯C11	3.138 (3)	C14⋯H7^vi^	2.97 (2)
C9⋯C20	3.472 (3)	H8⋯H11^vi^	2.53 (3)
C15⋯C19	3.450 (3)	H9⋯H11	2.58 (3)
C2⋯H5	2.476 (18)		

Symmetry codes: (i) 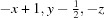; (ii) 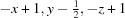; (iii) 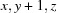; (iv) 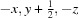; (v) 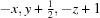; (vi) 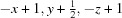.

**Table 3:** Calculated energies and other parameters for (I)

Parameter	Value in (I)
Total energy *E*_tot_ (eV)	−30168.2025
*E*_HOMO_ (eV)	−6.6964
*E*_LUMO_ (eV)	−0.8090
Energy gap, *ΔE* (eV)	5.8878
Dipole moment, *μ* (Debye)	2.5919
Ionization potential, *I* (eV)	6.6964
Electron affinity, *A*	0.8090
Electro negativity, *χ*	4.0554
Hardness, *η*	2.9437
Electrophilicity index, *ω*	2.3920
Softness, *σ*	0.3397
Fraction of electrons transferred, *ΔN*	0.5516

**Table 4:** Comparison of the selected (X-ray and DFT) geometric data (Å, °)

Bonds/angles	X-ray	B3LYP/6–311G(d,p)
O1—C2	1.203 (3)	1.237
O2—C3	1.208 (2)	1.242
N2—C3	1.346 (3)	1.379
N2—C1	1.472 (3)	1.494
N2—C19	1.454 (3)	1.470
N1—C3	1.399 (3)	1.414
N1—C2	1.360 (3)	1.384
N1—C16	1.453 (3)	1.467
C3—N2—C1	112.90 (16)	112.45
C3—N2—C19	121.81 (18)	120.14
N2—C3—N1	107.02 (17)	106.95
C3—N1—C16	122.9 (2)	122.80
C2—N1—C3	112.60 (17)	112.41
O2—C3—N2	128.0 (2)	127.88
O2—C3—N1	125.0 (2)	125.10

**Table 5:** Experimental details

Crystal data	
Chemical formula	C_21_H_16_N_2_O_2_
*M*_r_	328.36
Crystal system, space group	Monoclinic, *P*2_1_
Temperature (K)	296
*a*, *b*, *c* (Å)	10.144 (3), 7.952 (2), 10.928 (3)
β (°)	97.104 (12)
*V* (Å^3^)	874.8 (4)
*Z*	2
Radiation type	Mo *K*α
μ (mm^−1^)	0.08
Crystal size (mm)	0.34 × 0.17 × 0.12

Data collection	
Diffractometer	Bruker APEXII CCD
Absorption correction	Multi-scan (*SADABS*; Bruker, 2016 ▸)
*T*_min_, *T*_max_	0.694, 0.746
No. of measured, independent and observed [*I* > 2σ(*I*)] reflections	21744, 3988, 3529
*R*_int_	0.037
(sin θ/λ)_max_ (Å^−1^)	0.650

Refinement	
*R*[*F* ^2^ > 2σ(*F* ^2^)], *wR*(*F* ^2^), *S*	0.035, 0.090, 1.03
No. of reflections	3988
No. of parameters	290
No. of restraints	1
H-atom treatment	All H-atom parameters refined
Δρ_max_, Δρ_min_ (e Å^−3^)	0.14, −0.16
Absolute structure	Flack *x* determined using 1436 quotients [(*I* ^+^)−(*I* ^−^)]/[(*I* ^+^)+(*I* ^−^)] (Parsons et al., 2013 ▸)
Absolute structure parameter	−0.3 (4)

Computer programs: *APEX3* and *SAINT* (Bruker, 2016 ▸), *SHELXT* (Sheldrick, 2015*a* ▸), *SHELXL* (Sheldrick, 2015*b* ▸) and *OLEX2* (Dolomanov *et al.*, 2009 ▸).

## supplementary crystallographic information

### Crystal data

**Table:**

C_21_H_16_N_2_O_2_	*F*(000) = 344
*M**_r_* = 328.36	*D*_x_ = 1.247 Mg m^−^^3^
Monoclinic, *P*2_1_	Mo *K*α radiation, λ = 0.71073 Å
*a* = 10.144 (3) Å	Cell parameters from 9652 reflections
*b* = 7.952 (2) Å	θ = 3.2–27.2°
*c* = 10.928 (3) Å	µ = 0.08 mm^−^^1^
β = 97.104 (12)°	*T* = 296 K
*V* = 874.8 (4) Å^3^	Prism, colourless
*Z* = 2	0.34 × 0.17 × 0.12 mm

### Data collection

**Table:**

Bruker APEXII CCD diffractometer	3529 reflections with *I* > 2σ(*I*)
φ and ω scans	*R*_int_ = 0.037
Absorption correction: multi-scan (SADABS; Bruker, 2016)	θ_max_ = 27.5°, θ_min_ = 1.9°
*T*_min_ = 0.694, *T*_max_ = 0.746	*h* = −13→13
21744 measured reflections	*k* = −10→10
3988 independent reflections	*l* = −13→14

### Refinement

**Table:**

Refinement on *F*^2^	Hydrogen site location: difference Fourier map
Least-squares matrix: full	All H-atom parameters refined
*R*[*F*^2^ > 2σ(*F*^2^)] = 0.035	*w* = 1/[σ^2^(*F*_o_^2^) + (0.0457*P*)^2^ + 0.0829*P*] where *P* = (*F*_o_^2^ + 2*F*_c_^2^)/3
*wR*(*F*^2^) = 0.090	(Δ/σ)_max_ < 0.001
*S* = 1.03	Δρ_max_ = 0.14 e Å^−^^3^
3988 reflections	Δρ_min_ = −0.16 e Å^−^^3^
290 parameters	Absolute structure: Flack *x* determined using 1436 quotients [(*I*^+^)-(*I*^-^)]/[(*I*^+^)+(*I*^-^)] (Parsons et al., 2013)
1 restraint	Absolute structure parameter: −0.3 (4)
Primary atom site location: dual	

### Special details

**Table:**

Geometry. All esds (except the esd in the dihedral angle between two l.s. planes) are estimated using the full covariance matrix. The cell esds are taken into account individually in the estimation of esds in distances, angles and torsion angles; correlations between esds in cell parameters are only used when they are defined by crystal symmetry. An approximate (isotropic) treatment of cell esds is used for estimating esds involving l.s. planes.

### Fractional atomic coordinates and isotropic or equivalent isotropic displacement parameters (Å^2^)

**Table:**

	*x*	*y*	*z*	*U*_iso_*/*U*_eq_	
O1	0.27390 (15)	0.2664 (2)	0.19321 (15)	0.0502 (4)	
O2	−0.01151 (15)	0.7093 (2)	0.18041 (16)	0.0521 (4)	
N1	0.10965 (17)	0.4650 (2)	0.16652 (16)	0.0421 (4)	
N2	0.20794 (16)	0.6856 (2)	0.26052 (16)	0.0408 (4)	
C1	0.30827 (18)	0.5513 (3)	0.27974 (18)	0.0380 (4)	
C2	0.2324 (2)	0.4054 (3)	0.20923 (18)	0.0387 (4)	
C3	0.09114 (19)	0.6317 (3)	0.20165 (18)	0.0390 (4)	
C4	0.33794 (18)	0.5114 (2)	0.41722 (19)	0.0385 (4)	
C5	0.2604 (2)	0.3960 (3)	0.4718 (2)	0.0458 (5)	
C6	0.2784 (3)	0.3712 (4)	0.5987 (2)	0.0565 (6)	
C7	0.3738 (3)	0.4604 (4)	0.6713 (2)	0.0572 (6)	
C8	0.4520 (2)	0.5750 (3)	0.6180 (2)	0.0526 (6)	
C9	0.4342 (2)	0.6008 (3)	0.4921 (2)	0.0454 (5)	
C10	0.42895 (19)	0.5861 (3)	0.21163 (19)	0.0395 (4)	
C11	0.5495 (2)	0.5070 (3)	0.2474 (2)	0.0441 (5)	
C12	0.6546 (2)	0.5272 (3)	0.1782 (2)	0.0543 (6)	
C13	0.6410 (3)	0.6249 (4)	0.0733 (2)	0.0606 (6)	
C14	0.5225 (3)	0.7025 (4)	0.0372 (2)	0.0622 (7)	
C15	0.4163 (2)	0.6834 (3)	0.1052 (2)	0.0538 (6)	
C16	0.0113 (3)	0.3724 (4)	0.0851 (2)	0.0541 (6)	
C17	−0.0433 (2)	0.2305 (3)	0.1448 (3)	0.0613 (7)	
C18	−0.0849 (4)	0.1125 (5)	0.1895 (5)	0.1046 (14)	
C19	0.2236 (3)	0.8536 (3)	0.3129 (2)	0.0504 (5)	
C20	0.1674 (3)	0.8738 (4)	0.4290 (3)	0.0701 (8)	
C21	0.1218 (5)	0.8918 (10)	0.5191 (5)	0.136 (2)	
H5	0.189 (2)	0.328 (3)	0.420 (2)	0.051 (7)*	
H6	0.228 (3)	0.296 (4)	0.632 (3)	0.068 (8)*	
H7	0.389 (3)	0.445 (4)	0.763 (3)	0.080 (10)*	
H8	0.522 (3)	0.636 (4)	0.669 (2)	0.059 (7)*	
H9	0.486 (3)	0.677 (4)	0.455 (3)	0.075 (9)*	
H11	0.562 (2)	0.435 (4)	0.323 (2)	0.054 (7)*	
H12	0.736 (3)	0.474 (4)	0.203 (3)	0.069 (8)*	
H13	0.720 (3)	0.641 (4)	0.027 (3)	0.068 (8)*	
H14	0.511 (3)	0.777 (4)	−0.037 (3)	0.072 (8)*	
H15	0.330 (3)	0.737 (4)	0.076 (3)	0.060 (7)*	
H16A	−0.051 (3)	0.446 (4)	0.056 (3)	0.060 (8)*	
H16B	0.053 (3)	0.332 (4)	0.014 (3)	0.070 (9)*	
H18	−0.113 (6)	0.010 (8)	0.228 (6)	0.18 (2)*	
H19A	0.315 (3)	0.879 (4)	0.328 (2)	0.061 (7)*	
H19B	0.182 (3)	0.926 (4)	0.250 (3)	0.067 (8)*	
H21	0.087 (5)	0.905 (8)	0.590 (5)	0.16 (2)*	

### Atomic displacement parameters (Å^2^)

**Table:**

	*U*^11^	*U*^22^	*U*^33^	*U*^12^	*U*^13^	*U*^23^
O1	0.0528 (9)	0.0399 (8)	0.0585 (10)	0.0036 (7)	0.0088 (7)	−0.0076 (7)
O2	0.0430 (8)	0.0499 (9)	0.0606 (10)	0.0084 (7)	−0.0045 (7)	0.0041 (7)
N1	0.0425 (9)	0.0389 (9)	0.0426 (9)	−0.0019 (7)	−0.0033 (7)	−0.0008 (8)
N2	0.0373 (8)	0.0349 (9)	0.0492 (10)	0.0009 (7)	0.0014 (7)	−0.0011 (7)
C1	0.0361 (9)	0.0342 (9)	0.0432 (11)	0.0012 (8)	0.0035 (8)	0.0012 (8)
C2	0.0414 (10)	0.0385 (10)	0.0368 (10)	−0.0003 (8)	0.0070 (8)	0.0009 (8)
C3	0.0396 (10)	0.0403 (11)	0.0365 (10)	0.0013 (8)	0.0024 (8)	0.0058 (8)
C4	0.0349 (9)	0.0385 (11)	0.0423 (11)	0.0052 (8)	0.0055 (8)	0.0012 (8)
C5	0.0414 (11)	0.0468 (12)	0.0501 (12)	0.0006 (9)	0.0094 (9)	0.0032 (10)
C6	0.0574 (13)	0.0592 (15)	0.0556 (14)	0.0074 (12)	0.0183 (11)	0.0142 (12)
C7	0.0644 (15)	0.0677 (16)	0.0400 (12)	0.0230 (13)	0.0082 (10)	0.0047 (11)
C8	0.0531 (12)	0.0545 (14)	0.0476 (13)	0.0104 (11)	−0.0037 (10)	−0.0093 (11)
C9	0.0438 (11)	0.0440 (11)	0.0478 (12)	0.0016 (9)	0.0035 (9)	−0.0013 (9)
C10	0.0386 (9)	0.0391 (11)	0.0412 (10)	−0.0022 (8)	0.0060 (7)	0.0009 (8)
C11	0.0405 (10)	0.0450 (12)	0.0468 (12)	0.0014 (9)	0.0054 (9)	0.0040 (10)
C12	0.0419 (11)	0.0616 (15)	0.0605 (15)	0.0032 (11)	0.0105 (10)	0.0011 (12)
C13	0.0519 (13)	0.0735 (17)	0.0601 (14)	−0.0040 (12)	0.0219 (11)	0.0055 (13)
C14	0.0692 (16)	0.0698 (17)	0.0498 (14)	−0.0003 (13)	0.0166 (12)	0.0179 (13)
C15	0.0482 (12)	0.0622 (15)	0.0507 (13)	0.0044 (11)	0.0053 (10)	0.0129 (12)
C16	0.0540 (13)	0.0525 (14)	0.0520 (14)	−0.0059 (11)	−0.0086 (11)	−0.0047 (12)
C17	0.0463 (12)	0.0490 (14)	0.088 (2)	−0.0035 (10)	0.0067 (12)	−0.0068 (13)
C18	0.081 (2)	0.066 (2)	0.174 (4)	−0.0128 (18)	0.043 (2)	0.017 (2)
C19	0.0479 (13)	0.0358 (11)	0.0664 (15)	0.0013 (9)	0.0025 (11)	−0.0020 (11)
C20	0.0587 (15)	0.0758 (19)	0.0742 (19)	0.0032 (14)	0.0024 (13)	−0.0289 (16)
C21	0.103 (3)	0.214 (6)	0.094 (3)	0.001 (4)	0.026 (2)	−0.069 (4)

### Geometric parameters (Å, º)

**Table:**

O1—C2	1.203 (3)	C10—C11	1.388 (3)
O2—C3	1.208 (2)	C10—C15	1.390 (3)
N1—C2	1.360 (3)	C11—C12	1.391 (3)
N1—C3	1.399 (3)	C11—H11	1.00 (3)
N1—C16	1.453 (3)	C12—C13	1.377 (4)
N2—C1	1.472 (3)	C12—H12	0.94 (3)
N2—C3	1.346 (3)	C13—C14	1.366 (4)
N2—C19	1.454 (3)	C13—H13	1.01 (3)
C1—C10	1.534 (3)	C14—H14	1.00 (3)
C2—C1	1.545 (3)	C15—C14	1.390 (4)
C4—C1	1.529 (3)	C15—H15	0.99 (3)
C4—C5	1.390 (3)	C16—H16A	0.89 (3)
C4—C9	1.390 (3)	C16—H16B	0.98 (3)
C5—C6	1.389 (3)	C17—C16	1.447 (4)
C5—H5	1.02 (3)	C17—C18	1.162 (5)
C6—C7	1.370 (4)	C18—H18	0.97 (6)
C6—H6	0.90 (3)	C19—C20	1.462 (4)
C7—H7	1.01 (3)	C19—H19A	0.94 (3)
C8—C7	1.384 (4)	C19—H19B	0.95 (3)
C8—H8	0.98 (3)	C20—C21	1.148 (5)
C9—C8	1.380 (3)	C21—H21	0.90 (5)
C9—H9	0.93 (3)		
			
O1···H16B	2.84 (2)	C20···C4	3.372 (3)
O1···H13^i^	2.61 (2)	C20···C9	3.472 (3)
O1···H5	2.767 (18)	C2···H5	2.476 (18)
O1···H8^ii^	2.62 (2)	C4···H11	2.679 (18)
O2···H18^iii^	2.68 (5)	C6···H9^ii^	2.97 (2)
O2···H19B	2.65 (2)	C6···H18^v^	2.90 (4)
O2···H16A	2.50 (2)	C8···H11^vi^	2.94 (2)
O2···H16B^iv^	2.33 (2)	C8···H19A^ii^	2.83 (2)
N2···H15	2.53 (2)	C9···H11	2.73 (2)
C4···C20	3.372 (3)	C10···H9	2.75 (2)
C9···C19	3.378 (3)	C10···H19A	2.96 (2)
C9···C11	3.138 (3)	C11···H14^i^	2.94 (2)
C9···C20	3.472 (3)	C11···H9	2.79 (2)
C11···C9	3.138 (3)	C12···H14^i^	2.91 (2)
C15···C19	3.450 (3)	C14···H7^vi^	2.97 (2)
C19···C15	3.450 (3)	H8···H11^vi^	2.53 (3)
C19···C9	3.378 (3)	H9···H11	2.58 (3)
			
C2—N1—C3	112.60 (17)	C8—C9—H9	121.2 (19)
C2—N1—C16	124.3 (2)	C11—C10—C1	120.65 (18)
C3—N1—C16	122.9 (2)	C11—C10—C15	118.4 (2)
C3—N2—C1	112.90 (16)	C15—C10—C1	120.64 (18)
C3—N2—C19	121.81 (18)	C10—C11—C12	120.2 (2)
C19—N2—C1	124.78 (17)	C10—C11—H11	120.6 (14)
N2—C1—C2	100.41 (15)	C12—C11—H11	119.3 (14)
N2—C1—C4	109.84 (16)	C11—C12—H12	119.8 (18)
N2—C1—C10	112.27 (16)	C13—C12—C11	120.9 (2)
C4—C1—C2	111.07 (16)	C13—C12—H12	119.3 (18)
C4—C1—C10	116.25 (15)	C12—C13—H13	119.5 (17)
C10—C1—C2	105.76 (16)	C14—C13—C12	119.3 (2)
O1—C2—N1	126.3 (2)	C14—C13—H13	121.2 (17)
O1—C2—C1	126.93 (19)	C13—C14—C15	120.6 (2)
N1—C2—C1	106.74 (16)	C13—C14—H14	121.1 (17)
O2—C3—N1	125.0 (2)	C15—C14—H14	118.2 (17)
O2—C3—N2	128.0 (2)	C10—C15—C14	120.7 (2)
N2—C3—N1	107.02 (17)	C10—C15—H15	119.9 (16)
C5—C4—C1	120.41 (18)	C14—C15—H15	119.3 (16)
C9—C4—C1	120.74 (18)	N1—C16—H16A	107.0 (19)
C9—C4—C5	118.6 (2)	N1—C16—H16B	108.8 (17)
C4—C5—H5	121.0 (14)	C17—C16—N1	113.0 (2)
C6—C5—C4	120.7 (2)	C17—C16—H16A	112.0 (18)
C6—C5—H5	118.4 (14)	C17—C16—H16B	108.8 (18)
C5—C6—H6	119 (2)	H16A—C16—H16B	107 (2)
C7—C6—C5	120.0 (2)	C18—C17—C16	177.3 (4)
C7—C6—H6	120.8 (19)	C17—C18—H18	176 (3)
C6—C7—C8	119.8 (2)	N2—C19—C20	114.0 (2)
C6—C7—H7	121.8 (18)	N2—C19—H19A	108.9 (18)
C8—C7—H7	118.3 (18)	N2—C19—H19B	104.8 (18)
C7—C8—H8	120.2 (16)	C20—C19—H19A	107.8 (16)
C9—C8—C7	120.4 (2)	C20—C19—H19B	112.0 (17)
C9—C8—H8	119.3 (16)	H19A—C19—H19B	109 (2)
C4—C9—H9	118.4 (19)	C21—C20—C19	178.8 (5)
C8—C9—C4	120.4 (2)	C20—C21—H21	179 (4)
			
C3—N2—C1—C4	−112.10 (13)	C2—N1—C3—N2	4.70 (16)
C19—N2—C1—C4	59.82 (18)	C16—N1—C3—N2	−170.68 (14)
C3—N2—C1—C10	116.91 (13)	C9—C4—C5—C6	0.3 (2)
C19—N2—C1—C10	−71.17 (19)	C1—C4—C5—C6	−173.66 (14)
C3—N2—C1—C2	4.97 (15)	C4—C5—C6—C7	−0.3 (3)
C19—N2—C1—C2	176.88 (13)	C5—C6—C7—C8	0.0 (3)
C9—C4—C1—N2	−87.33 (15)	C9—C8—C7—C6	0.3 (3)
C5—C4—C1—N2	86.52 (16)	C4—C9—C8—C7	−0.3 (3)
C9—C4—C1—C10	41.52 (19)	C5—C4—C9—C8	0.0 (2)
C5—C4—C1—C10	−144.63 (14)	C1—C4—C9—C8	173.96 (14)
C9—C4—C1—C2	162.49 (13)	N2—C1—C10—C11	159.40 (13)
C5—C4—C1—C2	−23.66 (18)	C4—C1—C10—C11	31.74 (19)
O1—C2—C1—N2	177.04 (14)	C2—C1—C10—C11	−92.03 (15)
N1—C2—C1—N2	−1.91 (14)	N2—C1—C10—C15	−27.33 (19)
O1—C2—C1—C4	−66.81 (19)	C4—C1—C10—C15	−154.99 (14)
N1—C2—C1—C4	114.23 (13)	C2—C1—C10—C15	81.25 (17)
O1—C2—C1—C10	60.15 (19)	C15—C10—C11—C12	0.6 (2)
N1—C2—C1—C10	−118.80 (12)	C1—C10—C11—C12	174.00 (15)
C3—N1—C2—O1	179.51 (14)	C10—C11—C12—C13	−0.2 (3)
C16—N1—C2—O1	−5.2 (2)	C11—C12—C13—C14	−0.1 (3)
C3—N1—C2—C1	−1.52 (16)	C12—C13—C14—C15	0.0 (3)
C16—N1—C2—C1	173.78 (14)	C10—C15—C14—C13	0.4 (3)
C19—N2—C3—O2	2.4 (2)	C11—C10—C15—C14	−0.7 (3)
C1—N2—C3—O2	174.63 (14)	C1—C10—C15—C14	−174.13 (17)
C19—N2—C3—N1	−178.28 (14)	C2—N1—C16—C17	69.8 (2)
C1—N2—C3—N1	−6.09 (16)	C3—N1—C16—C17	−115.34 (18)
C2—N1—C3—O2	−175.99 (14)	C3—N2—C19—C20	76.6 (2)
C16—N1—C3—O2	8.6 (2)	C1—N2—C19—C20	−94.61 (19)

Symmetry codes: (i) −*x*+1, *y*−1/2, −*z*; (ii) −*x*+1, *y*−1/2, −*z*+1; (iii) *x*, *y*+1, *z*; (iv) −*x*, *y*+1/2, −*z*; (v) −*x*, *y*+1/2, −*z*+1; (vi) −*x*+1, *y*+1/2, −*z*+1.

### Hydrogen-bond geometry (Å, º)

*Cg*1 and *Cg*2 are the centroids of the C4–C9 and C10–C15 rings, respectively.

**Table:**

*D*—H···*A*	*D*—H	H···*A*	*D*···*A*	*D*—H···*A*
C16—H16*B*···O2^vii^	0.98 (3)	2.33 (3)	3.178 (3)	144 (2)
C9—H9···*Cg*1^vi^	0.93 (3)	2.93 (2)	3.778 (2)	152.7 (17)
C14—H14···*Cg*2^viii^	1.00 (3)	2.87 (2)	3.762 (3)	149.6 (17)

Symmetry codes: (vi) −*x*+1, *y*+1/2, −*z*+1; (vii) −*x*, *y*−1/2, −*z*; (viii) −*x*+1, *y*+1/2, −*z*.
